# Investigation of bimodal characteristics of the droplet size distribution in condensation spray

**DOI:** 10.1038/s41598-023-39087-5

**Published:** 2023-07-25

**Authors:** Junnosuke Okajima, Mitsuki Kato, Akihiro Hayakawa, Yuka Iga

**Affiliations:** 1 Institute of Fluid Science, Tohoku University, Sendai, 980-8579 Japan; 2 Mechanical Engineering Division, Tohoku University, Sendai, 980-8579 Japan

**Keywords:** Mechanical engineering, Fluid dynamics

## Abstract

To understand the generation process of airborne droplets during exhalation, this study investigates the mechanism of bimodal characteristics of the size distribution of droplets generated in a condensed spray flow. The phase change process in the condensed spray flow was estimated based on the droplet size distribution measured by a phase Doppler particle analyzer and the temperature distribution measured by a thermistor. On the central axis, the size distribution was unimodal in the spray interior. In contrast, bimodality of the size distribution at the outer edge of the spray flow was observed. At the edge of the spray flow, a large temperature gradient was formed. This indicates that condensation actively occurred at the outer edge. For the same reason as outlined above, condensation did not progress at the spray center because of the consumption of water vapor at the outer edge by the condensation, and the droplet diameter did not change significantly. Hence, owing to the difference in the local phase change process between the center and outer edge of the spray, large and small droplets can exist simultaneously in the middle region. As a result, the size distribution of the condensation spray is bimodal.

## Introduction

COVID-19 was first identified in the Chinese city of Wuhan in late 2019 and has since spread worldwide. It continues to mutate and ravage the world. It is feasible and reasonable measures to avoid “airtight,” “dense,” and “close” at the stage when the countermeasure has not been established, not only for the novel coronavirus infectious viral disease but also for any unknown air/droplet infection. The countermeasure for “airtightness” is ventilation of space and air purification. This technology can contribute to mechanical engineering and fluid engineering. Wei and Li^1^ summarized the aerosol infection in the indoor environment. They mentioned that the range of transmission routes depends on the droplet’s diameter. Hence, the transportation of droplets by the airflow generated in the indoor environment is essential to design and evaluating the ventilation system. When designing the arrangement of ventilation systems, air purification systems, and sterilizers, the diffusion path and airborne period of droplets should be predicted based on theory and numerical analysis. In response to this novel coronavirus, researchers in the field of computational fluid dynamics worldwide are conducting numerical analyses of droplets. Stiehl et al.^2^ conducted a numerical simulation of transportation and evaporation of droplets discharged by sneezing. Oh et al. carried out a numerical fluid analysis of indoor ventilation^3^ and numerically compared the removal efficiency of droplets and droplet nuclei caused by coughing between mechanical ventilation and natural ventilation. The research group led by Tsubokura also numerically reproduced the scattering of droplets and aerosols and the effects of ventilation in various indoor environments^4^ and outdoor barbecues^5^, by a large-scale numerical analysis using Fugaku^6^. Furthermore, droplet transportation was simulated in many situations, such as the classroom^7^, restaurant^8^, urban bus^9^, airplane^10^, and air-conditioning system^11^.

In the analysis of computational fluid dynamics, the motion of droplets was tracked in a Lagrangian manner using an equation of motion that considers the aerodynamic force based on the relative velocity with the surrounding air for each droplet. The velocity and position of droplets were obtained by tracking time^5^. For droplets generated from the mouth, the time variation of the flow rate and droplet size distribution of phonation and cough are given as boundary conditions based on the measured data from experiments. As an example of droplet models, Bale et al. used a speaking model^4^. In addition, there are several droplet models based on actual measurements, such as a cough model^12,13^, sneeze model^14^, a case in which the mouth is open and closed even with a cough^14^, conversation at normal volume, and conversation in a loud voice^5^. As shown in these models, the variations in the droplet number and flow rate are affected by the language, speaking form, and individual differences, and the difference is significantly dependent on the literature. To perform an analysis with computational fluid dynamics that matches the actual phenomena more closely, more measured data should be collected, and a database should be built with model droplets that are more in-depth, such as generation of droplets in the respiratory tract^15^.

Droplets that transmit infection are released from the respiratory tract by respiratory activities, such as coughing, sneezing, talking, and breathing. It is considered that respiratory droplets are generated by the following mechanisms: shear-induced surface-wave instability in the airway lining fluid^16^, oral cavity mode^17^, film rupture in the bronchiolar branch^18^. The time scales of single droplet was estimated by the combination of evaporation and falling behavior by gravity^19,20^. The physico-chemical characteristics influencing to the virus in a droplet during evaporation was evaluated by Vejerano and Marr^21^. Studies above were focused the behavior of single droplet, however, the generated droplets have a broad size distribution with diameters ranging from less than one micrometers to several hundred micrometers. By using such data, de Oliveira et al.^22^ evaluated the viral activity in the droplet by using model of mass transfer by evaporation. Johnson et al.^23^ measured the size distribution of aerosols generated during coughing and talking. In addition, Morawska et al.^24^ measured the both of size distribution and concentration of droplets during expiratory activities. Almstrand et al.^25^ measured exhaled particles by using optical particle counter in the range of 0.3–2.0 $\upmu \hbox {m}$. Lindsley et al. also measured the size distribution of aerosols generated by coughing and talking. They reported that the average number of droplets generated was 75,400 at the onset of the disease and 52,200 at the insurgence of the disease^26^. Asadi et al. reported that about 1–50 droplets are generated per second depending on the volume of voice just by talking, even if the respiratory activity is not as intense as coughing or sneezing and is not visible^27^. Regarding the size characteristics of aerosols, including SARS-CoV-2, Liu et al. investigated a hospital in Wuhan City, China, where mass infection occurred and reported peaks in 0.25–1.0 $\upmu \hbox {m}$ and 1 $\upmu \hbox {m}$ or more^28^.

As described in several cases above, although there are differences due to coughing, sneezing, conversation, language, and individual differences in droplets discharged from the mouth, a common feature is that the size distribution of droplets is bimodal^5,28^. In contrast, it is well known that in a spray flow in which liquid is discharged from a nozzle at a high speed and is formed by the atomization of liquid caused by the gas–liquid interface instability, there is a single peak in the size distribution of atomized droplets^29–31^. It can be considered that the droplet size distribution is bimodal because the atomization and evaporation/condensation processes are affected by several factors such as shape of the oral cavity, unsteady discharge flow rate, viscosity of saliva, colloid effect of a solid foreign matter such as bacteria, and temperature and pressure differences between the body and outside air. Among these elementary processes, it is unknown about the phase change, especially the formation and growth of droplets by condensation. As exhaled air contains a large amount of water vapor, understanding the process by which water vapor condenses in the air may help understand aerosol formation. Since the droplet growth by the condensation develops in space, it is essential to evaluate the spatial change of the droplet diameter and the particle size distribution.

Therefore, in this study, a fundamental experiment of spray flow generated by the condensation of water steam was carried out with a focus on the evaporation and condensation processes. Especially the spatial variation of droplet characteristics was focused on. In the condensation nozzle, high-temperature and high-pressure steam is discharged into the atmosphere at room temperature, and mist is formed by the condensation accompanied by a decrease in temperature. For the condensation spray flow, the size distribution of the droplet diameter and the flow velocity were measured using a phase Doppler particle analyzer (PDPA). The spatial temperature distribution was also measured. Based on the measurement data, the formation mechanism of bimodality of the size distribution was discussed.

## Methods

**Figure 1:**
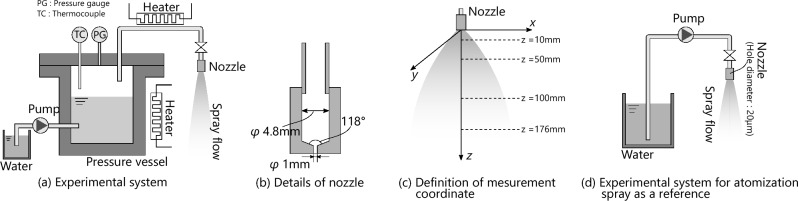
Descriptions of experimental systems and definition of measurement points.

Figure 1a shows a schematic of the condensed spray-generation system. The experimental system consisted of a pressure tank, a spray nozzle, a plunger pump, and measurement devices. The pressure tank stored water at temperatures greater than 100 $^{\circ }\hbox {C}$, and the flow rate was controlled by operating a valve installed between the pressure tank and the spray nozzle. A plunger pump was used to transfer water at 90 $^{\circ }\hbox {C}$into the pressure tank. The pressure tank was heated using a heater equipped with a temperature controller. In addition, the transfer pipe between the pressure tank and the spray nozzle was heated by the heater to prevent the condensation of steam inside the pipe and nozzle. Figure 1b shows the cross-sectional view of the spray nozzle. The inner diameter of the inlet was 4.8 mm in diameter, and the channel was contracted by a cone shape with a 118 $^{\circ }$ apex angle into 1 mm in diameter.

The pressure tank was filled with distilled water and heated to 120 $^{\circ }\hbox {C}$. The steam was discharged into the atmosphere by opening the valve connected to the nozzle. The discharged steam expanded and cooled after passing through the nozzle, and a droplet was formed by condensation. In this study, the droplet size distribution, droplet velocity, and temperature were measured as characteristics of the spray flow. Figure 1c shows the definition of the coordinates and measurement points.

Figure 1d shows the experimental setup for the atomization spray. The spray produced by this system is an outflow of high-pressure liquid that breaks up into droplets because of the liquid instability. The atomization process of the condensation spray is discussed by comparing the results of condensation and atomization. The nozzle diameter was 20 $\upmu \hbox {m}$, and distilled water was sprayed at 10 MPa and 10 mL / min.

### PDPA

A PDPA is a noncontact measurement method that enables the simultaneous measurement of the diameter and one-directional velocity of each droplet while passing a droplet in an inspection volume formed by two laser beams. The measurement system produced by Dantec Dynamics Co. Ltd. was used. An Ar laser of 514.5 nm was utilized. In each spatial location, 10,000 droplets were sampled at each spatial location. For statistical analysis, 85% percentile was adapted to filter out outliers. The Sauter mean diameter was evaluated as the representative value of the droplet size distribution, as expressed by the following equation:

1 $$\begin{aligned} D_{32} = \frac{\displaystyle {\sum n_i d_i^3}}{\displaystyle {\sum n_i d_i^2}}, \end{aligned}$$

where $d_i$ denotes the droplet diameter and $n_{i}$ represents the number of droplets with the diameter $d_i$.

### Temperature measurement

The local temperature inside the spray flow was measured using a thermistor probe (N317 /BR14KA103K /23300 /RPS /3 /SP, Nikkiso-Thermo Co., Ltd.). The thermistor chip is enclosed with epoxy in a polyimide tube, and the outer diameter of the sensing position is 0.6 mm. The electrical resistance of the thermistor was measured using a digital multimeter(DMM4040,Tektronix Co., Ltd.). The spatial position of the thermistor probe was adjusted using an X-Z stage.

## Results

### Droplet characteristics

**Figure 2:**
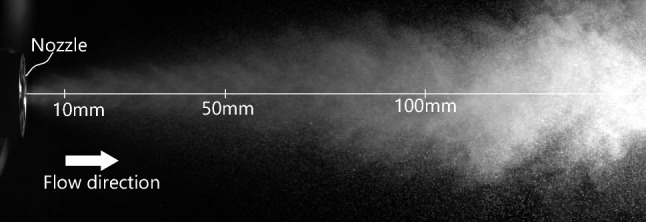
Photograph of condensation spray.

**Figure 3:**
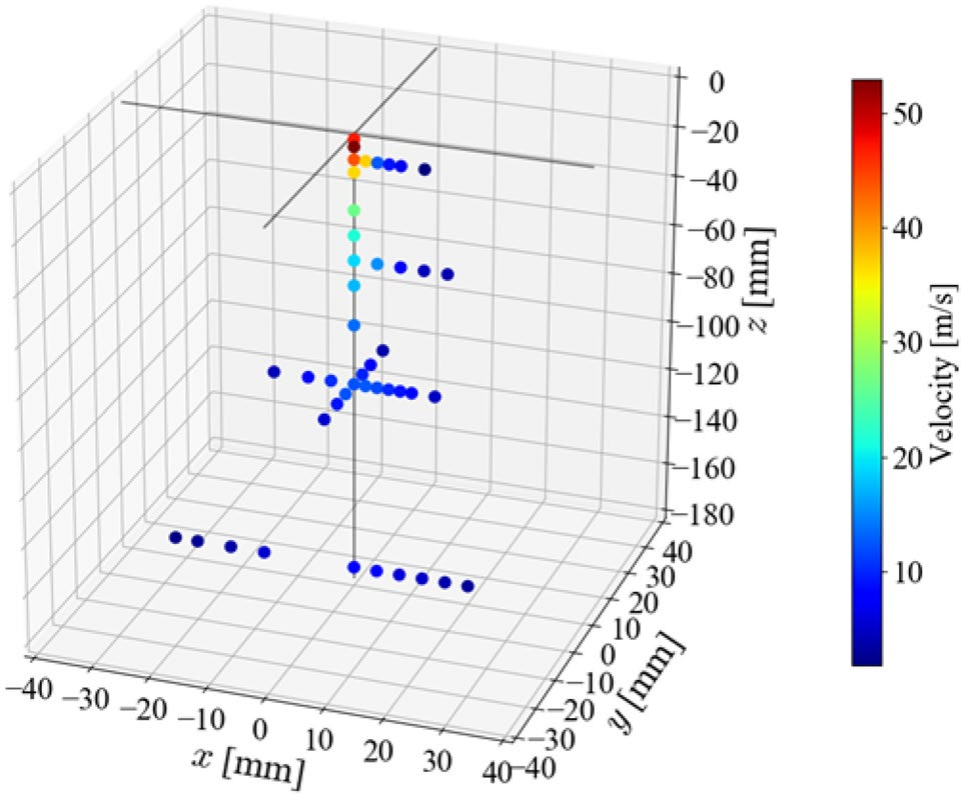
Axis directional velocity distribution of the condensed spray flow.

Figure 2 shows a snapshot of the condensation spray, and Fig. 3 shows the example of spatial distribution of the average velocity on the *z*-direction. The axial velocity varies from 53.0 m/s at the exit of the nozzle to 4.75 m/s, which is the terminal velocity.

**Figure 4:**
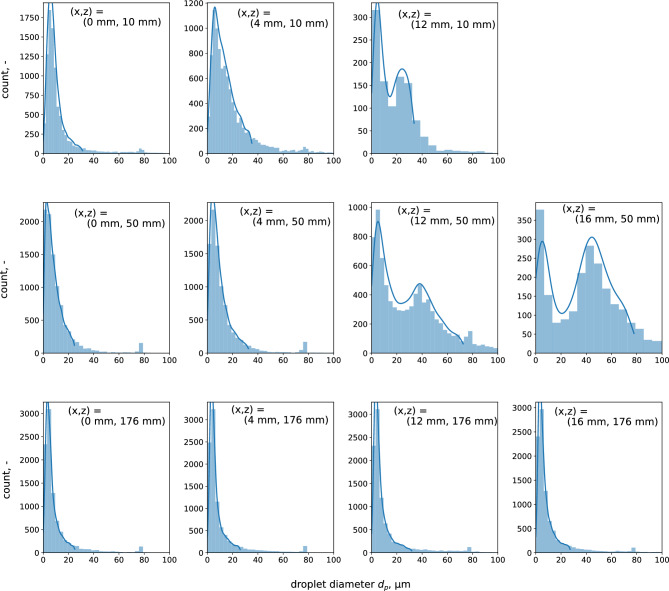
Size distributions of the condensed spray flow at spatial specific points.

Figure 4 shows the size distribution of the droplets in the spray flow at each spatial position. Here, small peaks around 80 $\upmu \hbox {m}$ are observed in almost all distributions. These peaks correspond to the specific bias error in the used PDPA system. The bars represent the droplet count for each diameter, and the line is determined by the kernel density estimation with a Gaussian kernel.

First, at *x* = 0 mm, the shape of the distribution does not change at any *z*, and droplets smaller than 20 $\upmu \hbox {m}$ are dominant. Furthermore, at *z* = 176 mm, which is sufficiently downstream, similar distributions are found at all *x* locations. In other words, the size distribution of the spray is uniform in the sufficiently downstream space.

In contrast, the measured values of position (*x*, *z*) = (12 mm, 10 mm), (12 mm, 50 mm), and (16 mm, 50 mm) at the outer edge of the spray show a bimodal size distribution. In addition to droplets of 20 $\upmu \hbox {m}$ or less, which are observed at other points, a distribution with a peak around 30–50 $\upmu \hbox {m}$ is formed.

**Figure 5:**
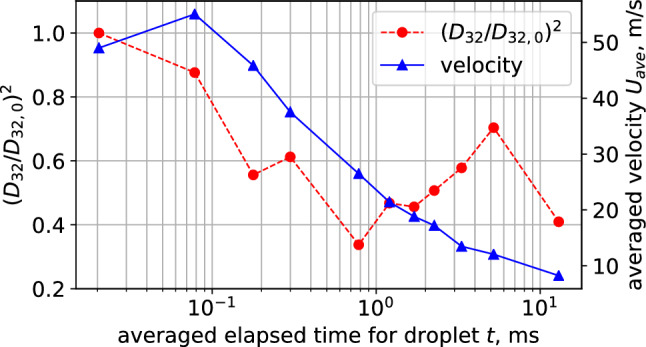
Time variation of averaged droplet surface area and axial velocity on the central axis.

Figure 5 shows the evaporation timescale and averaged axial velocity on the central axis. The averaged elapsed time of droplet at the position *z* is calculated as following equation.

2 $$\begin{aligned} t(z) = \int _{0}^{z} \frac{dz'}{U_{ave}(z')}. \end{aligned}$$

The ordinate of Fig. 5 is the normalized surface area based on the initial droplet. As shown in Fig. 5, the surface area of droplets linearly decrease till 1 ms and the trend follows the $d^2$ law^32^.

The average surface area decreased almost 0.6 at the early stage and it increased after 1 ms. Afterward, it decreased again to a value of around 0.4.

**Figure 6:**
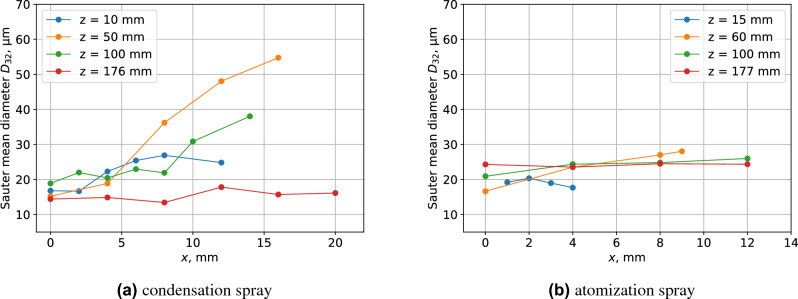
Spatial distribution of the Sauter mean diameter.

Figure 6a shows the distribution of the Sauter mean diameter of the condensed spray along the x-axis. In addition, the general liquid atomization nozzle results produced by the experiment (Fig. 1d) are shown in Fig. 6b to compare the atomization process. The spray produced by this nozzle is an outflow of high-pressure liquid that breaks up into droplets because of the liquid instability. In the case of condensed spray, the droplet diameter at the outer edge of the spray has a significant maximum value at a point 50 mm from the *z*-axis, and then decreases toward the downstream. At *z* = 176 mm, the Sauter mean diameter becomes uniform in the *x*-direction, as in the previous results. However, in the case of atomized spray, the Sauter mean diameter does not change much in space, as compared with that of the condensed spray.

### Temperature distribution

**Figure 7:**
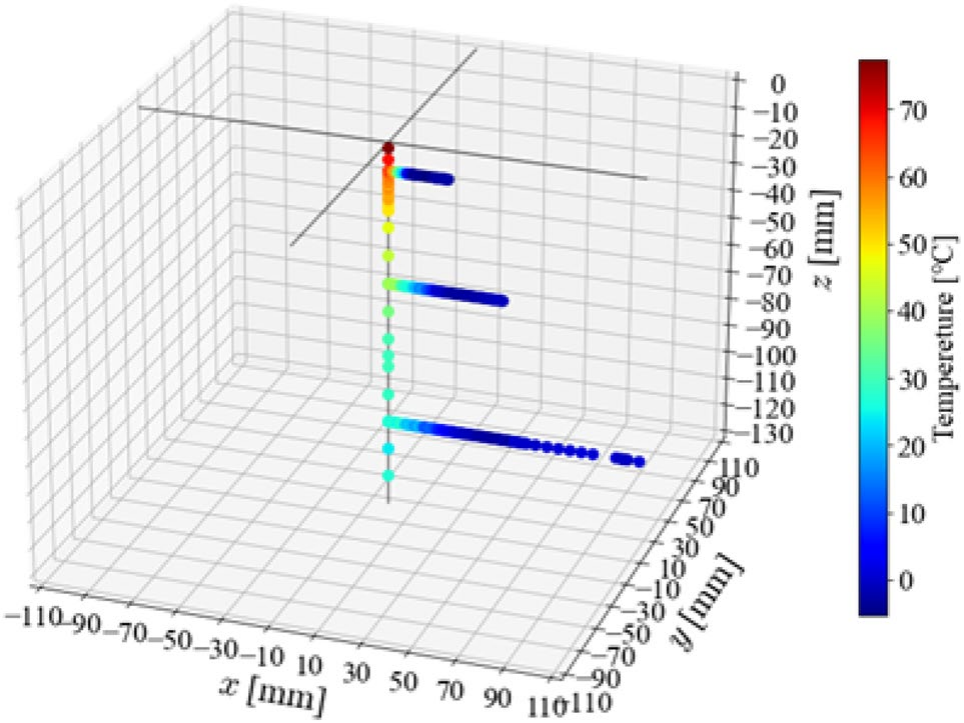
Spatial distribution of the temperature variation to ambient temperature in the condensation spray flow.

Figure 7 shows the spatial temperature distribution of a condensed spray flow. Here, the temperature difference with respect to ambient temperature is shown. Because the steam inside the nozzle is 120 $^{\circ }\hbox {C}$, the temperature is high immediately after spraying, but it drops rapidly. On the *z*-axis, the temperature gradually decreases, whereas the temperature around the spray rapidly decreases to about room temperature. Next, the temperature distributions in the *x*- and *z*-directions are examined in detail.

**Figure 8:**
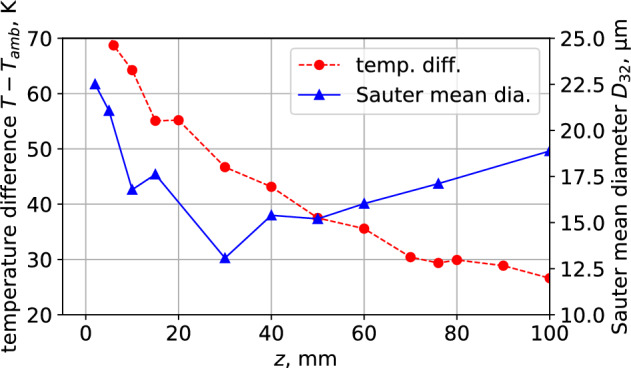
Axis directional velocity distribution of the condensed spray flow.

Figure 8 shows the temperature variation from the nozzle outlet to 100 mm downstream of the *z*-axis. The change in the Sauter mean diameter calculated in Fig. 6a is also plotted. It can be observed that the temperature decreases monotonically along the *z*-axis. In contrast, the Sauter mean diameter decreases to an extreme value up to 100 mm downstream and then increases.

**Figure 9:**
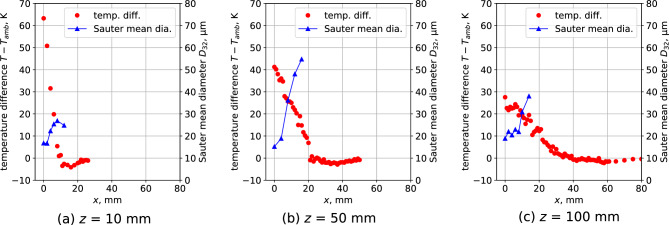
Axis directional velocity distribution of the condensed spray flow.

Figure 9 shows the radial temperature distributions at 10, 50, and 100 mm along the *z*-axis. The spatial distribution of the Sauter mean diameters shown in Fig. 6a is also plotted. On the central axis of the spray at all positions, the temperature is the highest, and the Sauter mean diameter is the lowest. It can be observed that the Sauter mean diameter increases with decreasing temperature in the *x*-direction. At a significantly farther position in the *x*-direction, a region where the temperature is lower than the room temperature is formed.

## Discussion

As described in the introduction, previous research has suggested that the size distribution of droplets generated during expiration has bimodal characteristics. In this study, as shown in Fig. 4, bimodality was observed in the size distribution at the positions of (12 mm, 10 mm), (12 mm, 50 mm), and (16 mm, 50 mm) of the condensed spray flow. The formation of bimodality and the droplet growth process in the condensed spray flow are considered here.

**Figure 10:**
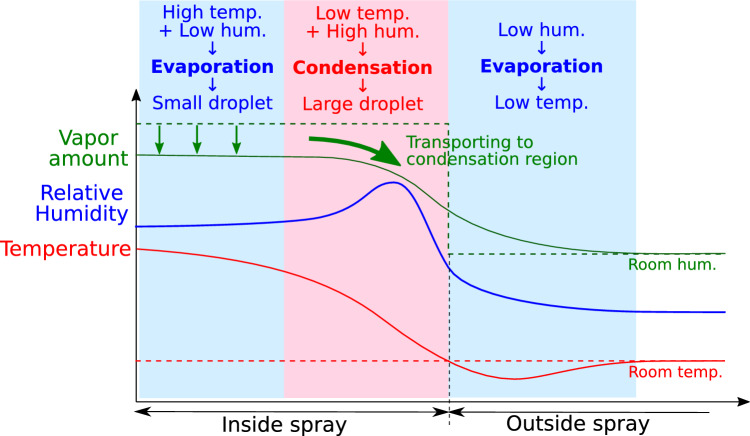
Schematic of the distribution of physical quantities on the radial direction to explain the formation mechanism of bimodality.

First, the formation of bimodality is discussed based on the variation in the physical quantities in the radial direction. Figure 10 shows an overview of this discussion. As shown in Fig. 10, the center of the condensed spray flow is at a high temperature, and the temperature decreases toward the outer edge. It is considered that the droplets and water vapor are cooled at the point where the temperature drops, and the droplets grow. Therefore, a large droplet corresponding to the second peak is generated in this region.

Because water vapor in the spray flow is actively consumed in this region owing to the growth of droplets, water vapor should be transported from the center region to the actively condensing region. In other words, the amount of water vapor required for droplet growth is insufficient around the condensation region. At the central position along the *z*-axis, the droplet remains small, as shown by the variation in the Sauter mean diameter in Figs. 5 and 8. The absence of droplet growth by condensation despite the decrease in temperature suggests that the humidity is not over-saturated in this region. For the variation along the *z*-axis, the Sauter mean diameter decreases slightly up to 30 mm downstream, and evaporation is possible here as shown in Fig. 5.

As shown in Fig. 9, at the foot of the temperature distribution, the lower temperature region than the room temperature is confirmed. It is considered that a temperature drop owing to the evaporation of droplets exists around this region. Here, the evaporation of droplets proceeds owing to the low humidity; as a result, the temperature decreases more than the ambient temperature. At *z* = 100 mm, the PDPA measurement was conducted up to *x* = 14 mm, and bimodality was not observed in the size distribution, as shown in Fig. 4. However, as inferred from the temperature distribution, a bimodal condition may exist outside *x* = 14 mm. As shown in Fig. 4, the Sauter mean diameters were uniform in the *x*-axis direction 176 mm downstream. Although the temperature at *z* = 176 mm was not measured, it was assumed that excess water vapor had already been consumed by the droplet development owing to condensation, and evaporation was dominant.

**Figure 11:**
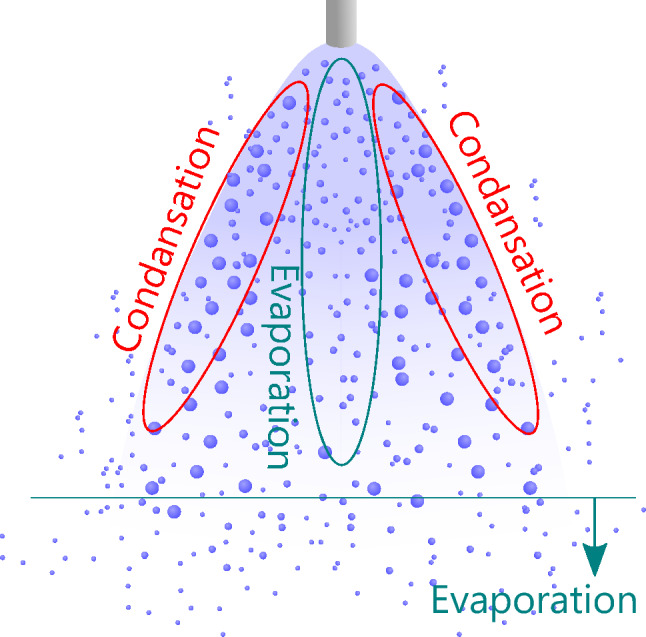
Schematic of the three-dimensional structure of the phase change region in the condensing spray flow.

Therefore, in the condensation spray flow, there are two regions inside: the spray outer edge, where droplets of a large diameter increase owing to condensation, and the spray center, where droplets of a small diameter increase owing to evaporation at high temperatures. The bimodality of the size distribution should be formed by adding both droplet size distributions. Figure 11 schematically shows the three-dimensional structure of the spray flow of condensed droplets. It is considered that evaporation is promoted at the center of the spray region because of the relatively low humidity caused by the flow of water vapor, and the condensation changes occur at the outer edge of the spray region, where the particle size is growing. The bimodality of the size distribution can be detected at a point located between the two regions.

Exhalation is accompanied by a complex vortex structure at the moment of exhalation, as shown in the expulsion process of coughing and sneezing presented by Gupta et al.^13^ When the droplets following the bimodal distribution generated with the process of condensation and evaporation shown in this study are transported by the complicated expiration flow, the bimodality in the space should be promoted more.

## Conclusions

This study investigated the mechanism of bimodal characteristics in the size distribution of droplets generated in exhaled air. The phase change process in a condensed spray flow was estimated based on the droplet size distribution and temperature distribution measurements using PDPA. The findings are as follows:

- On the central axis, a single peak was observed in the spray interior. Bimodality was observed in the size distribution at the outer edge of the spray flow. It indicates that condensation was active at the outer edge. In contrast, as the size distribution was spatially uniform at $z =$ 176 mm downstream of the spray, it is assumed that the droplets have reached an equilibrium state.
- In the upstream of the condensed spray flow, the temperature outside the spray was lower than the room temperature. In this region, it is considered that water vapor in the space is consumed in the above-mentioned region where condensation is active so that the relative humidity is lowered and the temperature is lowered due to the evaporation of droplets.
- For the same reason as outlined above, condensation did not progress at the spray center portion owing to the consumption of water vapor at the condensation portion at the outer edge, and the droplet diameter did not change significantly. Thus, droplets of almost 20 $\upmu \hbox {m}$ in diameter were maintained in this region.

This study should contribute to the understanding of aerosol formation by showing that bimodal size distribution occurs in droplet formation by condensation and evaporation and that it is caused by spatial variation of condensed spray.

## Data Availability

The datasets used and/or analysed during the current study available from the corresponding author on reasonable request.
